# The megascolecid earthworms (Annelida, Oligochaeta, Megascolecidae) in the Phu Quoc island, Vietnam, with descriptions of three new species

**DOI:** 10.3897/zookeys.932.50314

**Published:** 2020-05-12

**Authors:** Tung T. Nguyen, Dang H. Lam, Binh T. K. Trinh, Anh D. Nguyen

**Affiliations:** 1 Department of Biology, School of Education, Can Tho University, Can Tho City, Vietnam Can Tho University Can Tho Vietnam; 2 Department of Applied Biology, Faculty of Agriculture and Rural Development, Kien Giang University, Kien Giang, Vietnam Kien Giang University Kien Giang Vietnam; 3 Duy Tan University, 254, Nguyen Van Linh, Da Nang, Vietnam Duy Tan University Da Nang Vietnam; 4 Institute of Ecology and Biological Resources, Vietnam Academy of Science and Technology, 18, Hoangquocviet Rd., Caugiay District, Hanoi, Vietnam Institute of Ecology and Biological Resources, Vietnam Academy of Science and Technology Hanoi Vietnam

**Keywords:** biodiversity, taxonomy

## Abstract

The megascolecid earthworms of the Phu Quoc island are intensively investigated. Twelve species in three genera (*Lampito* Kinberg, 1867, *Amynthas* Kinberg, 1867, and *Metaphire* Sims & Easton, 1972) are recorded. Of these, *Metaphire doiphamon* Bantaowong & Panha, 2016 is recorded for the first time in Vietnam, and three species are newly described, namely *Amynthas catenatus***sp. nov.**, *A. phuquocensis***sp. nov.**, and *A. poropapillatus***sp. nov.** An identification key to 12 megascolecid species is provided as well.

## Introduction

Phu Quoc, located in the southernmost part of Vietnam, is the largest island in the country, with an area of 58,923 ha. It is covered largely by typically tropical forests. The earthworms of the Phu Quoc are poorly known with only seven species reported from the island. These include *Pontoscolex corethrurus* (Müller, 1857), *Metaphire californica* (Kinberg, 1867), *Metaphire peguana* (Rosa, 1890), and *Metaphire campanulata* (Rosa, 1890) (Thai et al. 2004). Three additional species, *Amynthas primadamae* (Michaelsen, 1934), *Amynthas tertiadamae* (Michaelsen, 1934), and *Amynthas alteradamae* (Michaelsen, 1934), have been erroneously recorded from Phu Quoc island (Thai et al. 2004; Nguyen et al. 2016, 2017), despite the fact that they were described from Poulo Dama (= Nam Du archipelago) (Michaelsen 1934).

To improve the knowledge of the earthworms of Vietnam, this study documents the diversity of the family Megascolecidae from Phu Quoc island; three new species are described.

## Material and methods

**Figure 1.**
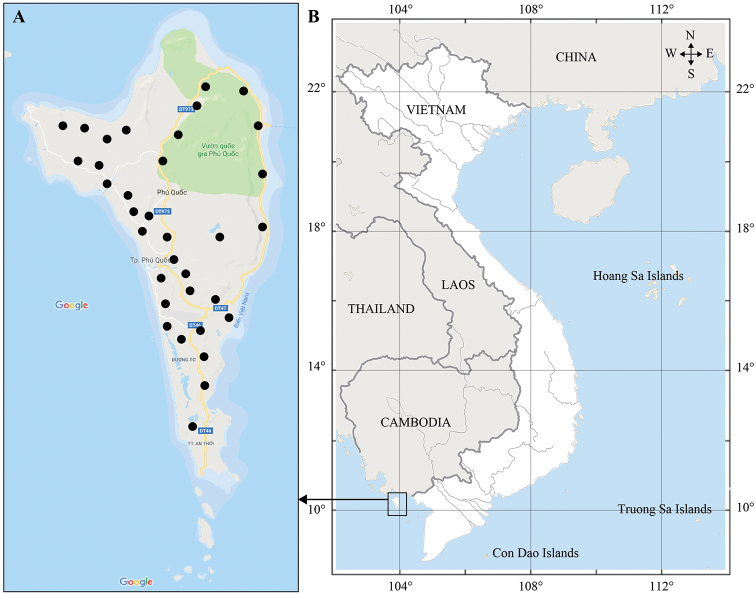
Collecting sites in the Phu Quoc island.

Earthworms were collected manually in 34 sites in three habitats: natural forests, industrial plantations of *Piper*, *Acacia*, and other trees, and residential gardens (Fig. 1). After their collection, specimens were killed using 2% formalin and fixed in 4% formalin for 24 hours and then transferred to new 4% formalin for morphological examination and long-term preservation.

Transverse body sections were processed using the classical method of haematoxylin and eosin. Selected segments were cleaned and dehydrated using graded ethanol concentrations. Segments were treated with paraffin, then cut using a Sakura Accu SRM 200CW microstome. The cut sections were stained using haematoxylin and eosin Y (Feldman and Wolfe 2014) and then transferred onto glass slides.

Material was examined under a Motic Digital microscope (model DM143-FBGG-C), and dissected from the dorsal side for internal observation. Colour images were taken using a camera attached directly to the microscope. Line drawings and colour images were improved and grouped into finished figures using Photoshop CS6.

All specimens including holotypes and paratypes are housed in the Laboratory of Zoology, Can Tho University. Some are shared with the laboratory of Department of Applied Biology, Kien Giang University.

**Abbreviations.**CTU = Can Tho University, ag = accessory glands, amp = ampulla, dv = diverticulum, gm = genital markings, mp = male pore, sp = spermathecal pore, ts = testis sacs, sv = seminal vesicles, ov = ovaries, cl = clitelum, ps = penial seta.

## Results

### Taxonomic account

#### Family Megascolecidae Rosa, 1891

##### Genus *Lampito* Kinberg, 1867

###### Lampito mauritii

Taxon classificationAnimaliaOpisthoporaMegascolecidae

Kinberg, 1867

F2DDD825-F8C4-5E01-8C96-8B08512C1066

Fig. 2

####### Material examined.

1 mature (CTU-EW.002.15), residential gardens (10°10'43"N, 103°58'24"E), 7 m, 5 November 2016, coll. Lam HD & Trinh TKB.

####### Diagnosis.

Medium-sized. Prostomium epilobous. Clitellum within xiv–xvii. Three pairs of spermathecal pores in ventral intersegments 6/7/8/9. No genital markings. Male pores in xviii with penis seta. Spermathecae with two diverticula. Holandric. Intestinal caeca absent. Septa 4/5/6 absent. Oesophageal gizzard within v–vi.

####### Remarks.

The species is widely distributed in sandy soils in coastal areas of Vietnam (Nguyen 2014; Nguyen et al. 2016).

####### Vietnamese name.

Giun lampito mauriti.

**Figure 2.**
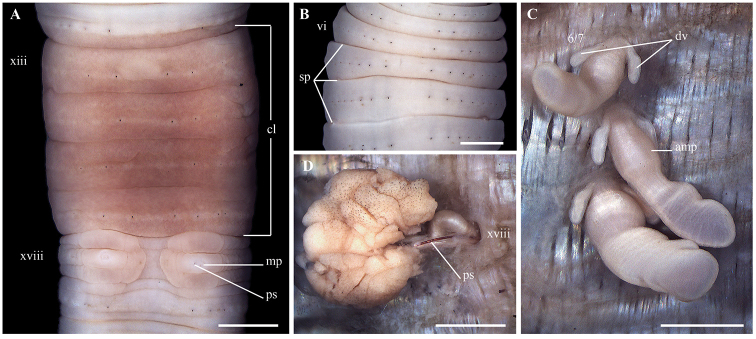
*Lampito mauritii* Kinberg, 1867 **A** Clitellum, ventral view **B** spermathecal region, ventral view **C** left spermatheca **D** Prostatic glands. Scale bar: 1mm.

##### Genus *Metaphire* Sims & Easton, 1972

###### Metaphire bahli

Taxon classificationAnimaliaOpisthoporaMegascolecidae

(Gates, 1945)

DDBCC598-1405-50F7-BF15-0B90323146C0

Fig. 3

####### Material examined.

22 matures (CTU-EW.004.46) natural forests, (10°10'48"N, 103°58'15"E), 20 m, 5 November 2016, coll. Lam HD & Trinh TKB; 30 matures (CTU-EW.004.47) residential gardens (10°11'06"N, 103°58'15"E), 13.4 m, 6 November 2016, coll. Lam HD & Trinh TKB; 13 matures (CTU-EW.004.48) industrial tree plantations, (10°06'11"N, 104°00'51"E), 20 m, 5 November 2016, coll. Lam HD & Trinh TKB.

####### Diagnosis.

Medium-sized. Prostomium 2/3 epilobous. First dorsal pore in 12/13. Three pairs of spermathecal pores in lateroventral intersegments 6/7/8/9. Male pores located inside copulatory pouches in xviii. Male region strongly concave to form an ellipsoid or rounded area. Genital markings absent in the spermathecal region, but two pairs present in 17/18 and 18/19, in line with male pores. Intestinal caeca simple. Holandric. Testis sacs separated. Septa 8/9/10 absent.

####### Habitats.

This species was collected from leaf litter and upper soil layer (0–10 cm).

**Remarks.** This species is widely distributed in southern Vietnam (Thai et al. 2004; Nguyen et al. 2016, 2017).

####### Vietnamese name.

Giun bahl.

**Figure 3.**
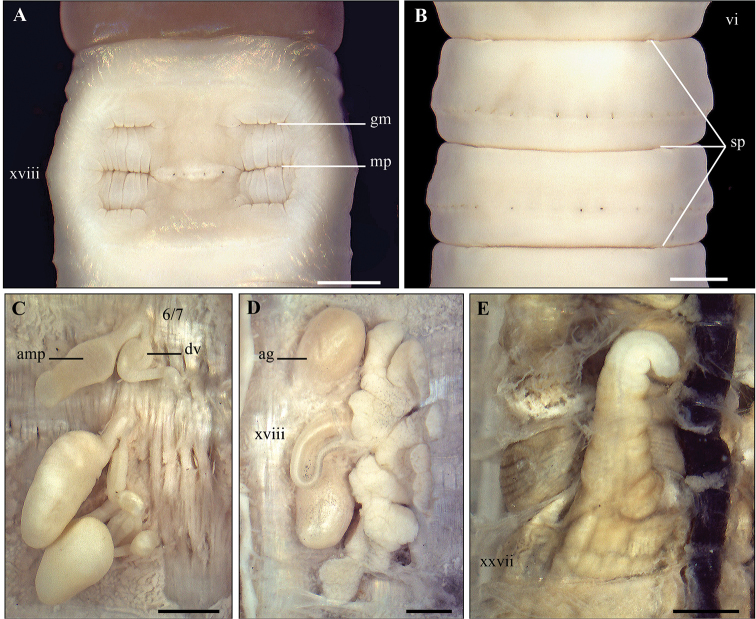
*Metaphire bahli* (Gates, 1945) **A** Male region, ventral view **B** spermathecal region, ventral view **C** left spermatheca **D** right prostatic gland **E** intestinal caecum; Scale bar: 1mm.

###### Metaphire californica

Taxon classificationAnimaliaOpisthoporaMegascolecidae

(Kinberg, 1867)

0761082F-3458-5355-BB8E-7AB4B688FB29

Fig. 4

####### Material examined.

21 matures (CTU-EW.136.02), residential gardens, (10°12'20"N, 103°57'53"E), 26 m, 6 November 2016, coll. Lam HD & Trinh TKB.

####### Diagnosis.

Medium-sized. Prostomium epilobous. First dorsal pore in 12/13. Two pairs of spermathecal pores in lateroventral intersegments 7/8/9. Male pores located inside copulatory pouches in xviii. Genital markings absent in both spermathecal and male regions. Intestinal caeca simple. Holandric. Testis sacs connected. Septa 8/9/10 absent.

####### Habitats.

The species was found in the residential gardens and natural forests. They live in leaf litter and humid rocks.

####### Remarks.

Thai et al. (2004) reported this species from the Phu Quoc island, but with question mark “*Pheretima californica*?”. Comparing to *M. californica* previously recorded in the mainland of Vietnam, the population of the Phu Quoc island has several differences: septum 10/11 absent, intestinal caeca simple, and testis sacs connected. However, simple intestinal caeca were also reported widely in Myanmar and Taiwan (Gates 1972; Chang et al. 2009).

####### Vietnamese name.

Giun california.

**Figure 4.**
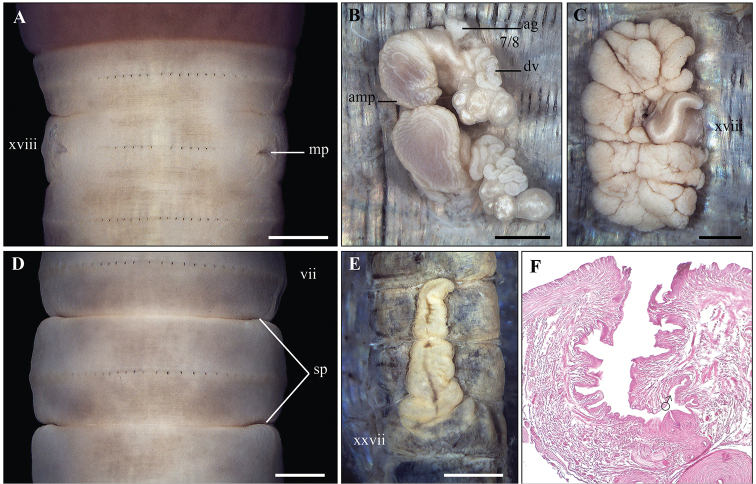
*Metaphire californica* (Kinberg, 1867) **A** Male region, ventral view **B** right spermatheca **C** right prostatic gland **D** spermathecal region **E** intestinal caecum **F** transection via copulatory pouch. Scale bar: 1mm.

###### Metaphire doiphamon

Taxon classificationAnimaliaOpisthoporaMegascolecidae

Bantaowong & Panha, 2016

BEAC577D-FE1A-5D65-B52E-9AC8172BA55C

Fig. 5

####### Material examined.

7 matures (CTU–EW.014.02), residential gardens (10°09'29"N, 104°00'01"E), 16 m, 5 November 2016, coll. Lam HD & Trinh TKB; 5 matures (CTU–EW.014.03), natural forests (10°21'08"N, 103°55'42"E), 82 m, 7 November 2016, coll. Lam HD & Trinh TKB.

**Diagnosis.** Medium-sized. Prostomium epilobous. First dorsal pore in 12/13. Three pairs of spermathecal pores in lateroventral intersegments 6/7/8/9. Male pores located inside copulatory pouches in xviii. Two pairs of genital markings in xvii and xix, in line with male pores. Intestinal caeca lobuled. Holandric. Testis sacs in xi, separated. Septa 8/9/10 absent.

####### Re-description.

Body cylindrical, medium-sized, length 168–220 mm, diameter 6.50–7.58 mm, segments 99–133, weight 5.5–13.2 g. Body greyish brown, ventrum paler than dorsum, clitellum darkish brown. Prostomium 3/4 epilobous. First dorsal pore in 12/13. Pre-clitellum setae stouter and sparser than post-clitellum setae; setal number: 65–77 in viii, 94–97 in xxx, 4–10 between two male porophores in xviii; setal distance: aa > ab, zz > zy. Clitellum within xiv–xvi, without setae and dorsal pores. Female pore single, in mid-ventral xiv.

Three pairs of spermathecal pores in lateroventral intersegments 6/7/8/9. Four pairs of pad-shaped genital markings in vi–ix, in line with spermathecal pores.

Male pores located inside copulatory pouches in xviii. Ventral distance between two openings of copulatory pouches about 0.3× body circumference. Two pairs of dish-shaped genital markings present in 17/18 and 18/19, in line with the openings of copulatory pouches.

Septa 5/6/7/8 thick, 8/9/10/11 absent, 11/12/13 thick. Oesophageal gizzard within viii–xi. Intestinal origin at xv. Intestinal caeca within xxvii–xxix, ventrally lobuled. Last hearts in xiii. Pharyngeal micronephridia grouped in 5/6/7. Typhlosole lamelliform. Lymph glands lobuled, from 16/17.

Spermathecae paired in vi–ix. Ampulla large, oval-shaped; ducts as long as 1/3 ampulla. Diverticula strongly coiled, attached to the middle of ampulla duct; seminal chamber oval. No accessory glands.

Holandric. Testis sacs in xi, separated. Seminal vesicles well developed in xi and xii. Ovaries in 12/13; ovisacs inviable. Prostate glands deeply lobuled in xvi–xxi; ducts spherical, enlarged basally. Two pairs of large accessory glands present.

####### Habitats.

The species was found in all habitats, but more in residential gardens. They live in clay soils in the depth of 15–30 cm.

####### Remarks.

Nguyen (2014) also recorded this species in the mountainous region of An Giang province under the name “*Metaphire* sp. 4”. Compared to the description of *M. doiphanon* by Bantaowong et al. (2016), our specimens differ slightly in the absence of septum 10/11, diverticula attached to the middle of ampulla duct, separated testis sacs, and intestinal caeca slightly lobuled ventrally. On the contrary, *M. doiphanon* has thick septum 10/11, diverticula attached to the base of ampulla duct, connected testis sacs, and simple intestinal caeca.

**Figure 5.**
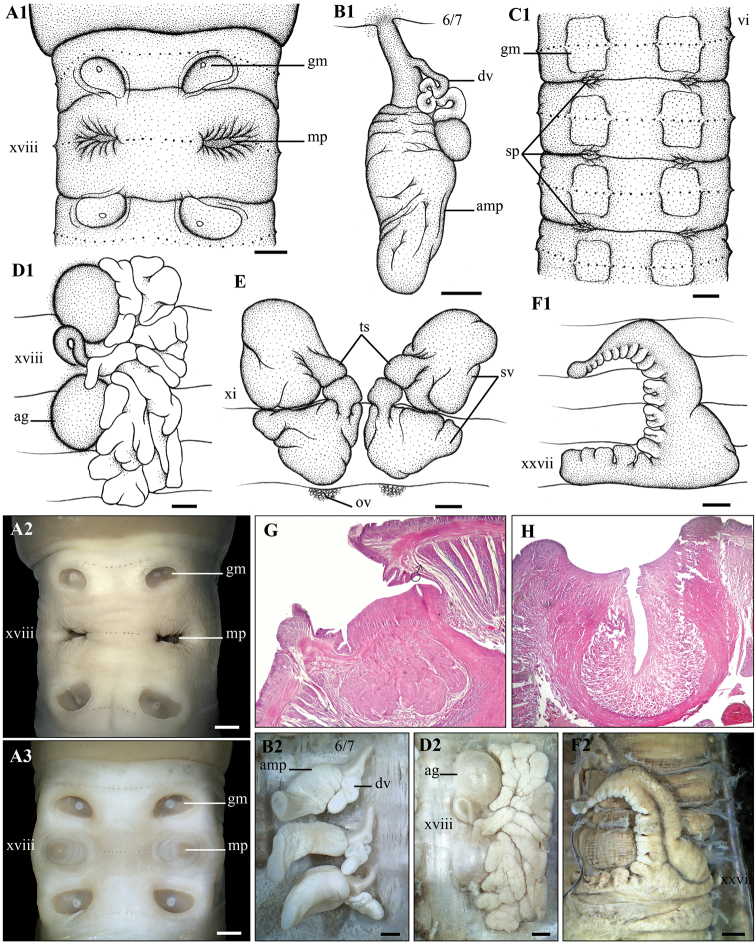
*Metaphire doiphamon* Bantaowong & Panha, 2016 **A1, A2, A3** Male region (concave form – **A2** and convex form – **A3**) ventral view **B1, B2** left spermatheca **C** spermathecal region, ventral view **D1, D2** right prostatic gland **E** testis sacs and vessciles **F1, F2** intestinal caecum **G** transection via male porophore **H** transection via genital markings. Scale bar: 1mm.

Of all specimens, five differ in that their copulatory pouches are deeply concave inside the body wall (Fig. 5A2), whereas, in others, the copulatory pouches are convex outside the body wall (Fig. 5A3).

###### Metaphire houlleti

Taxon classificationAnimaliaOpisthoporaMegascolecidae

(Perrier, 1872)

F1D6B755-8C45-5C93-A1AC-74BEA2E32E8E

Fig. 6

####### Material examined.

10 matures (CTU-EW.006.29), industrial tree plantations (10°06'11"N, 104°00'51"E), 20 m, 5 November 2016, coll. Lam HD & Trinh TKB; 15 matures (CTU-EW.018.024), residential gardens (10°16'04"N, 103°56'28"E), 6 November 2016, coll. Lam HD & Trinh TKB; 7 matures (CTU-EW.018.025), natural forests (10°22'53"N, 104°00'22"E), 38 m, 07 November 2016, coll. Lam HD & Trinh TKB.

####### Diagnosis.

Medium-sized. Prostomium epilobous. First dorsal pore in 11/12. Three pairs of spermathecal pores in lateroventral intersegments 6/7/8/9. Male pores located inside copulatory pouches in xviii. Two to four penial setae. Intestinal caeca simple. No genital markings. Holandric. Testis sacs connected. Septa 8/9/10 absent.

####### Habitats.

The species was found mainly in industrial tree plantations and residential gardens. It was collected in leaf litter and sometimes in upper soil surface (0–10 cm).

####### Remarks.

The specimens collected in Phu Quoc island have the seta a on segments iii–v being bigger than that on other segments.

####### Vietnamese name.

Giun houllet.

**Figure 6.**
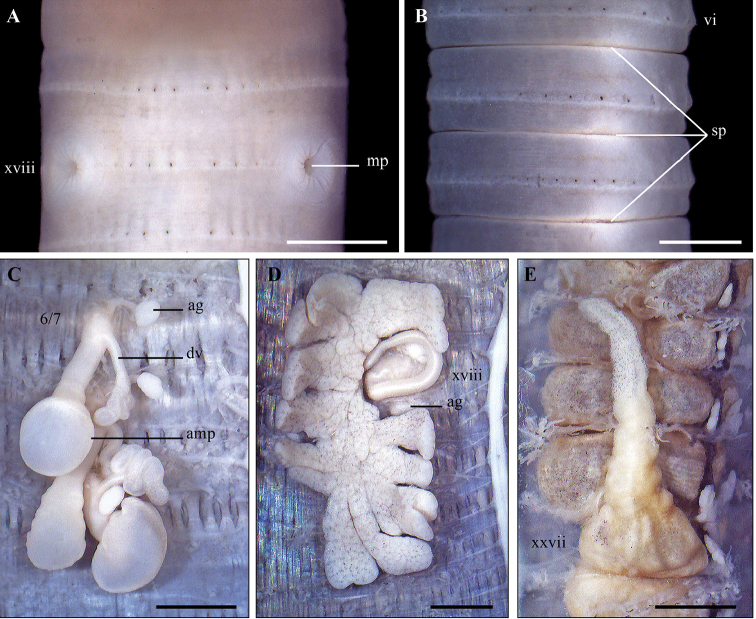
*Metaphire houlleti* (Perrier, 1872) **A** Male region, ventral view **B** spermathecal region, lateral view **C** left spermatheca **D** left prostatic gland **E** intestinal caecum. Scale bar: 1mm.

###### Metaphire peguana

Taxon classificationAnimaliaOpisthoporaMegascolecidae

(Rosa, 1890)

8F45FD3F-065E-57E3-9EE3-82A15E0DCCF1

Fig. 7

####### Material examined.

15 matures (CTU-EW.009.13) natural forests, (10°10'48"N, 103°58'15"E), 20 m, 5 November 2016, coll. Lam HD & Trinh TKB.

####### Diagnosis.

Medium-sized. Prostomium epilobous. First dorsal pore in 12/13. Three pairs of spermathecal pores in lateroventral intersegments 6/7/8/9. Male pores located inside copulatory pouches in xviii. Male region not concave. Genital markings absent in the spermathecal region, but two pairs disc-shaped in 17/18 and 18/19, in line with the openings of copulatory pouches. Intestinal caeca simple. Holandric. Testis sacs separated. Septa 8/9/10 absent.

####### Habitats.

The species was found only in the upper soil surface of natural forests.

####### Remarks.

Of 20 specimens, six have intestinal caeca which are slightly lobuled ventrally.

####### Vietnamese name.

Giun pegu.

**Figure 7.**
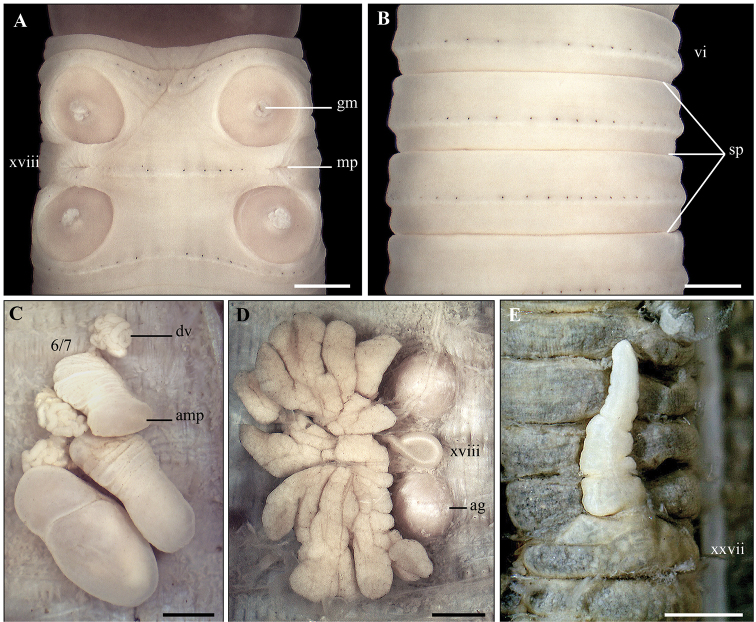
*Metaphire peguana* (Rosa, 1890) **A** Male region, ventral view **B** spermathecal region, ventral view **C** left spermathecae **D** left prostatic gland **E** intestinal caecum. Scale bar: 1mm.

###### Metaphire planata

Taxon classificationAnimaliaOpisthoporaMegascolecidae

(Gates, 1926)

FA5A8168-0088-5299-B995-B11C60BF5FDA

Fig. 8

####### Material examined.

10 matures (CTU-EW.005.030), industrial tree plantations (10°09'31"N, 104°00'38"E), 25 m, 5 November 2016, coll. Lam HD & Trinh TKB; 21 matures (CTU-EW.005.031), residential gardens (10°12'09"N, 103°58'06"E), 36 m, 5 November 2016, coll. Lam HD & Trinh TKB.

####### Diagnosis.

Medium-sized. Prostomium epilobous. First dorsal pore in 11/12. Two pairs of spermathecal pores in lateral intersegments 6/7/8. Male pores located inside copulatory pouches in xviii. Genital markings absent in the male region, but 1–3 located near each spermathecal pore. Intestinal caeca simple. Holandric. Testis sacs separated. Accessory glands present, sac-shaped. Septa 8/9/10 absent.

####### Habitats.

The species was commonly found in residential gardens and industrial tree plantations. They live in leaf litter and upper soil layer.

####### Vietnamese name.

Giun plana.

####### Remarks.

The species has been commonly found in southern Vietnam. However, it was erroneously identified as *Metaphire californica* (Nguyen 2014, as *Pheretima california*: sic!; Nguyen et al. 2016, 2017), but recently corrected (Nguyen et al. 2019). In addition, it has not been recorded from the small islands of Lai Son, An Son, and Hon Tre, which are located between Phu Quoc island and southern Vietnam (Nguyen et al. 2017).

**Figure 8.**
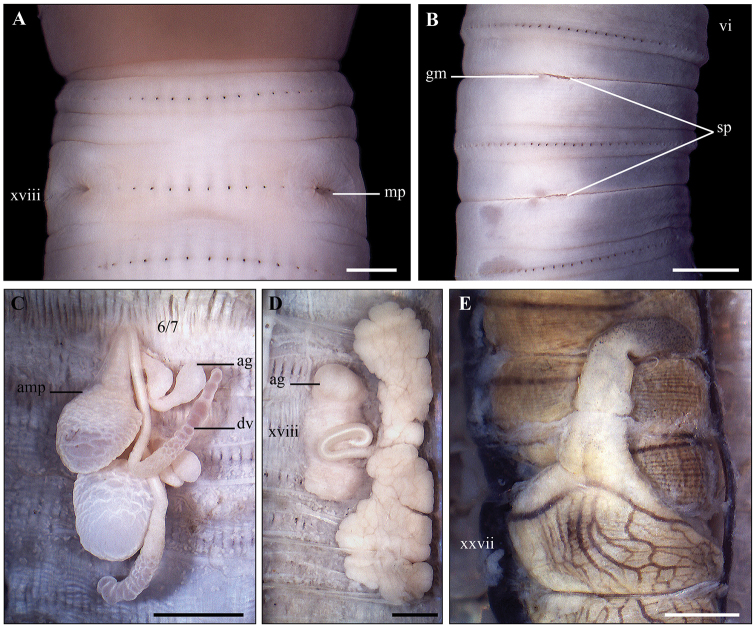
*Metaphire planata* (Gates, 1926) **A** Male region, ventral view **B** spermathecal region, lateral view **C** left spermathecae **D** right prostatic gland **E** intestinal caecum. Scale bar: 1mm.

###### Metaph ire posthuma

Taxon classificationAnimaliaOpisthoporaMegascolecidae

(Vaillant, 1868)

196E96B3-AFBD-5AC3-BA5C-50D3496168DA

Fig. 9

####### Material examined.

3 matures (CTU-EW.011.13), residential gardens (10°10'52"N, 103°58'08"E), 10 m, 5 November 2016, coll. Lam HD & Trinh TKB.

####### Diagnosis.

Medium-sized. Prostomium epilobous. First dorsal pore in 12/13. Four pairs of spermathecal pores in lateroventral intersegments 5/6/7/8/9. Male pores located inside shallow copulatory pouches in xviii. Two pairs of genital markings present in xvii and xix. Intestinal caeca simple. Holandric. Testis sacs connected. Septum 8/9 thick, 9/10 absent.

####### Habitats.

The species was only found in the residential gardens.

####### Vietnamese name.

Giun quắn.

####### Remarks.

The species is commonly distributed in southern Vietnam, but rarely found in Phu Quoc island.

**Figure 9.**
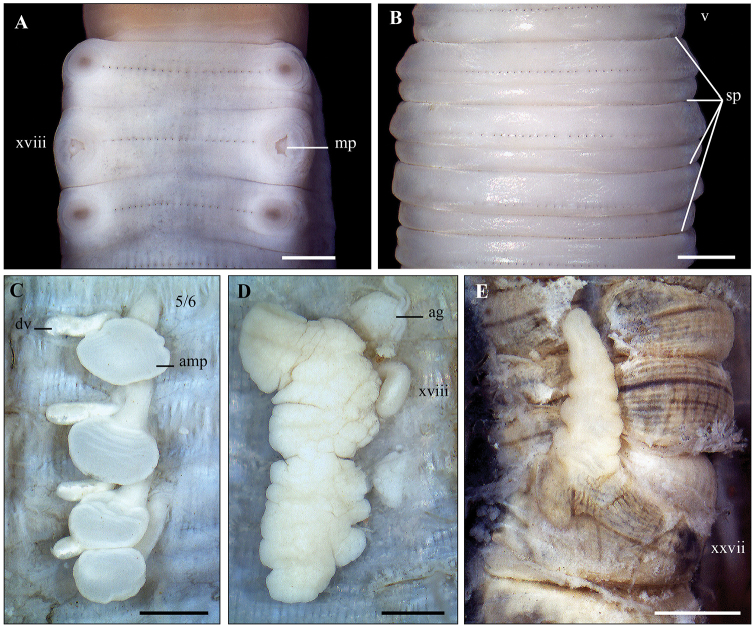
*Metaphire posthuma* (Vaillant, 1868) **A** Male region, ventral view **B** spermathecal region, ventral view **C** right spermatheca **D** left prostatic gland **E** intestinal caecum. Scale bar: 1mm.

###### Metaphire dorsobitheca

Taxon classificationAnimaliaOpisthoporaMegascolecidae

(Thai & Huynh, 1992)

FB172F8E-C3AD-5792-AFA1-C66770A30CD7

Fig. 10

####### Material examined.

1 mature (CTU–EW.191.h01), 9 matures (CTU-EW.191.p02), 24 matures (CTU-EW.191.03), Kien Giang Province, Phu Quoc island, industrial tree plantations (10°09'31"N, 104°00'38"E), 16 m, 5 November 2016, coll. Lam HD & Trinh TKB.

####### Diagnosis.

Small to medium-sized. Prostomium epilobous. First dorsal pore in 12/13. Three pairs of spermathecal pores in dorsal intersegments 6/7/8/9. Male pores located inside copulatory pouches in xviii. No genital markings. Penial setae present. Intestinal caeca simple. Holandric. Testis sacs connected in xi. Septa 8/9/10 absent.

####### Re-description.

Body cylindrical, small to medium-sized, length 81–118 mm, diameter 3.3–3.7 mm, segments 106–142, weight 0.5–0.8 g. Body uniformly whitish grey, clitellum darkish brown. Prostomium 1/2 epilobous. First dorsal pore in 12/13. Pre-clitellum setae stouter and sparser than post-clitellum setae; setal numbers: 44–47 in viii, 54–58 in xxx, 7–8 between two openings of copulatory pouches; setal distance aa > ab, zz ≥ zy. Clitellum within xiv–xvi, slightly flattened ventrally, without setae and dorsal pores. Female pore single, in mid-ventral xiv.

Three pairs of spermathecal pores in dorsal intersegments 6/7/8/9, located near dorsal line. Genital markings absent in the spermathecal region. Male pores located inside copulatory pouches in xviii; ventral distance between two openings of copulatory pouches about 0.3× body circumference. No genital markings in the male region.

Septa 5/6/7/8 thick, 8/9/10/11 absent, 11/12/13 thin. Oesophageal gizzard within viii–xi. Intestinal origin at xv. Intestinal caeca simple, within xxvii–xxv. Last hearts in xiii. Typhlosole lamelliform. Lymph glands lobuled, from 17/18.

Three pairs of spermathecae in vii–ix. Ampulla clavate; ducts long. Diverticula thin, waved, shorter than ampulla, attached to the base of ampulla; seminal chamber ellipsoid. No accessory glands.

Holandric. Testis sacs in xi, connected. Seminal vesicles xi and xii well developed. Ovaries in 12/13. Ovisacs invisible. Prostate glands racemose, within xvii–xxii; ducts spherical, enlarged basally, ending at bell-shaped copulatory pouches. Each copulatory pouch with a penial seta. Two pairs of accessory glands attached to copulatory pouches.

####### Habitats.

The species was collected in the depth of 0–10 cm of sandy soils in bushes and residential gardens.

####### Vietnamese name.

Giun buồng giao phối hình chuông.

####### Remarks.

Compared to the original description of *M. dorsobitheca* by Thai et al. (1992), our specimens differ in minor way: slightly larger size (length: 81–118mm vs 54 mm, diameter: 3.3–3.7 vs 2 mm), more segments (106–142 vs 76), first dorsal pore (12/13 vs 10/11), absence of septum 10/11 (vs presence), the origin of intestine (xv vs xvi), and shape of spermathecal ampulla (sac-shaped vs clavate).

*Metaphire dorsobitheca* was previously known only in its type locality (Dak No, Dak Lak Province). This is first time that this species has been found elsewhere.

**Figure 10.**
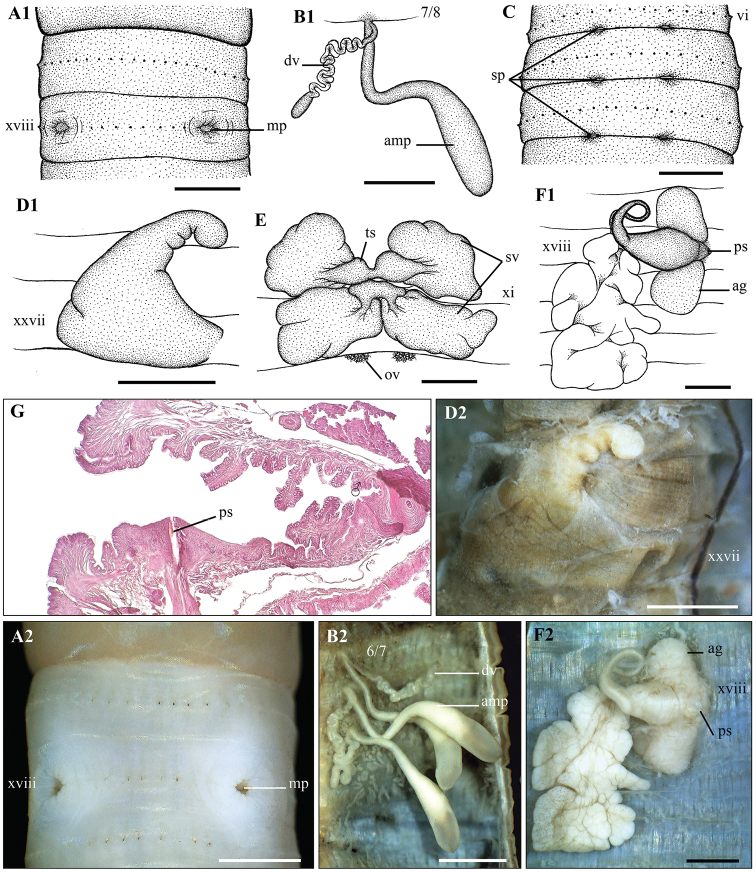
*Metaphire dorsobitheca* (Thai et Huynh, 1992) (CTU–EW.191.h01) **A1, A2** Male region, ventral view **B1, B2** right spermathecae **C** spermathecal region, dorsal view **D1, D2** intestinal caecum **E** Testis sacs and vesicles **F1, F2** left prostatic gland **G** transection via copulatory pouch. Scale bar: 1mm.

##### Genus *Amynthas* Kinberg, 1867

###### Amynthas catenatus sp. nov.

Taxon classificationAnimaliaOpisthoporaMegascolecidae

4F26309B-80C3-5222-A171-AA0CE35F26AB

http://zoobank.org/099A7863-3A99-45C7-B985-1B710D74371C

Fig. 11 , Table 1

####### Material examined.

***Holotype***: 1 mature (CTU-EW.189.h01), Kien Giang Province, Phu Quoc island, industrial tree plantations (10°06'11"N, 104°00'51"E), 20 m, 6 November 2016, coll. Lam HD & Trinh TKB.

***Paratypes***: 9 matures (CTU-EW.189.p02), same data as for holotype.

***Non-types***: 8 matures (CTU-EW.189.03), same data as for holotype.

####### Diagnosis.

Small to medium-sized, length 51–54 mm, diameter 2.2–2.5 mm, segments 85–89. Prostomium epilobous. Four pairs of spermathecal pores in lateroventral intersegments 5/6/7/8/9. Male pores in xviii, without copulatory pouches. Genital markings arranged in two transverse line in front of and behind the setal ring xviii. Intestinal caeca simple. Holandric. Testis sacs in xi and xii, separated. Septa 8/9/10 absent.

####### Etymology.

“*catenatus*”, an adjective in apposition, to emphasize the chained seminal chamber of diverticula.

####### Description.

Body cylindrical, small to medium-sized, length 51–54 mm, diameter 2.2–2.5 mm, segments 85–89, weight 1.2–1.4 g. Body uniformly whitish grey, clitellum darkish brown. Prostomium 1/2 epilobous. First dorsal pore in 5/6. Pre-clitellum setae stouter and sparser than post-clitellum setae; setal numbers: 47–54 in viii, 46–48 in xxx, no setae between two male porophores; setal distance: aa > ab, zz > zy. Clitellum within xiv–xvi, slightly flattened ventrally, without setae and dorsal pores. Female pore single, in mid-ventral xiv.

Four pairs of spermathecal pores in lateroventral intersegments 5/6/7/8/9. No genital markings in the spermathecal region.

Male porophores small in xviii, without copulatory pouches; ventral distance between two male pores about 0.25–0.28× body circumference. About 8–12 small genital markings ventrally arranged in two transverse lines in front of and behind the setal ring xviii.

Septa 5/6/7/8 thick, 8/9/10 absent, 10/11/12 thick. Oesophageal gizzard within viii–x. Intestinal origin at xv. Intestinal caeca simple, within xxvii–xxvi. Last hearts in xiii. Pharyngeal micronephridia in 5/6/7. Typhlosole lamelliform. Lymph glands absent.

Spermathecae paired in vi–ix. Ampulla clavate; ducts not clearly distinct from ampulla. Diverticula slightly waved, attached to the base of ampulla; seminal chamber small, chained-shaped with 2 or 3 parts. No accessory glands.

Holandric. Testis sacs in x and xi, connected. Seminal vesicles well developed in xi and xii. Ovaries in 12/13. Ovisacs invisible. Prostate glands deeply lobuled within xvi–xx; ducts U-shaped. No accessory glands.

####### Habitats.

The species was only collected under leaf litter in *Piper* plantations.

####### Vietnamese name.

Giun buồng tinh hình chuỗi.

####### Remarks.

The new species can be assigned to the *A. corticis* group, which is characterized by having four pairs of spermathecal pores in 5/6/7/8/9 and holandric (Sims and Easton 1972). This group has 108 described species worldwide, of which 17 have been recorded in Vietnam (Gates 1972; Sims and Easton 1972; Chang et al. 2009; Nguyen et al. 2016; Xiao 2019).

Compared to *A. corticis* (Kinberg, 1867), the new species differs in its smaller size (length 51–54 mm, diameter 2.2–2.5 mm vs length 45–170 mm, diameter 3.0–6.0 mm), first dorsal pore in 5/6 (vs in 11/12), and absence of pre-clitellar genital markings (vs genital markings paired near spermathecal pores). In addition, *A. catenatus* sp. nov. has 8–12 small genital markings ventrally arranged in two transverse lines in presetal and postsetal xviii, a clavate spermathecal ampulla, slightly wavy diverticula and attached to the base of ampulla, and a small seminal chamber, which is chained-shaped with 2 or 3 parts. On the contrary, *A. corticis* has two pairs of small circular genital markings located next to male pores in presetal and postsetal xviii, an ovoid ampulla, and a blunt, ovoid diverticula with straight stalk.

Within the *A. corticis* group, *Amynthas catenatus* sp. nov. is fairly similar to *A. divitopapillatus* (Thai, 1984) and *A. conhanungensis* (Thai, 1984) in having numerous genital markings in xviii and spermathecal pores in 5/6/7/8/9. However, they are differentiated in the position of first dorsal pore, number and arrangement of genital markings in the male region, presence of septum 8/9, type of intestinal caeca, and body size (summarized in Table 1).

**Figure 11.**
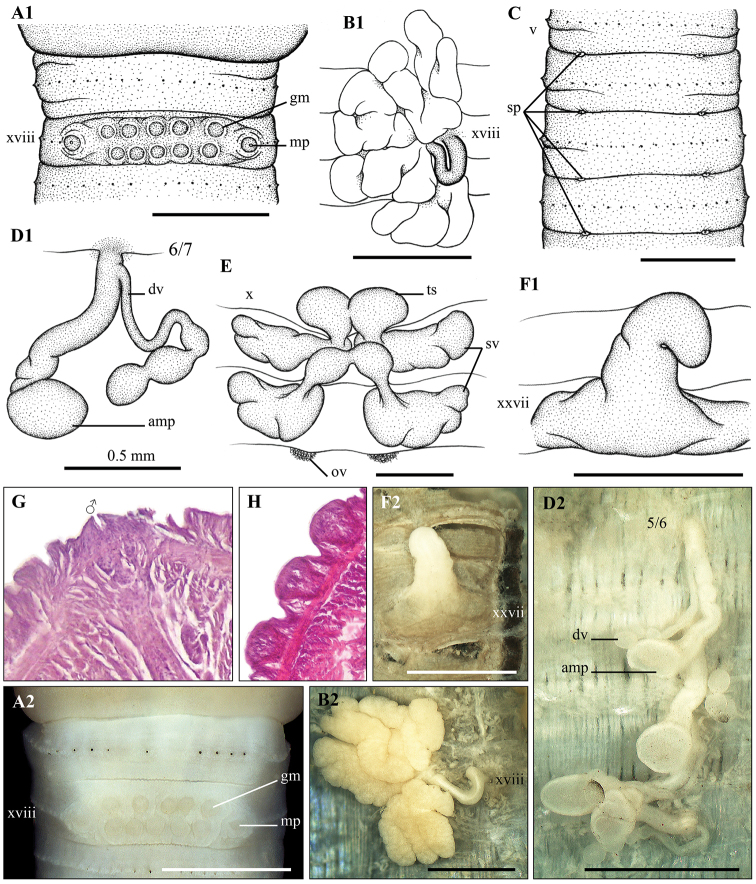
*Amynthas catenatus* sp. nov. Holotype CTU–EW.184.h01 **A1, A2** Male region, ventral view **B1, B2** left prostatic gland **C** spermathecal region, ventral view **D1, D2** left spermathecae **D** testis sacs and vesicles **F1, F2** intestinal caecum **G** transection via male porophore **H** transection via genital marking. Scale bar: 1mm.

**Table 1.** Character comparison between *A. catenatus* sp. nov., *A. conhanungensis* and *A. divitopapillatus*.

Characteristics	*A. catenatus*	*A. conhanungensis* ^1^	*A. divitopapillatus* ^1^
Length	51–54	220–350	121–126
Diameter	2.2–2.5	7–12	3–6
Segments	85–89	123–186	108–112
First dorsal pore	5/6	12/13	12/13
GM in spermathecal region	absent	numerous, vii–viii	numerous, vi–ix
GM in male region	8–10, two lines, pre- and post-setal in xviii	4–10, grouped around male pores in xviii	5–20, grouped around male pores in xviii
Septum 8/9	absent	thin	absent
Intestinal caeca	simple	lobuled	simple
Testis sacs	connected	separated	connected
Shape of ampulla	clavate	oval	oval
Seminal chamber	chained	twisted	chained

Source: ^1^ = Thai (1984).

###### Amynthas phuquocensis sp. nov.

Taxon classificationAnimaliaOpisthoporaMegascolecidae

BA7F0685-2F4C-5D53-8BD3-2FF8DBC149CF

http://zoobank.org/8165ADE3-0638-4DAF-8BA3-78092439F7B5

**Figure 12.**
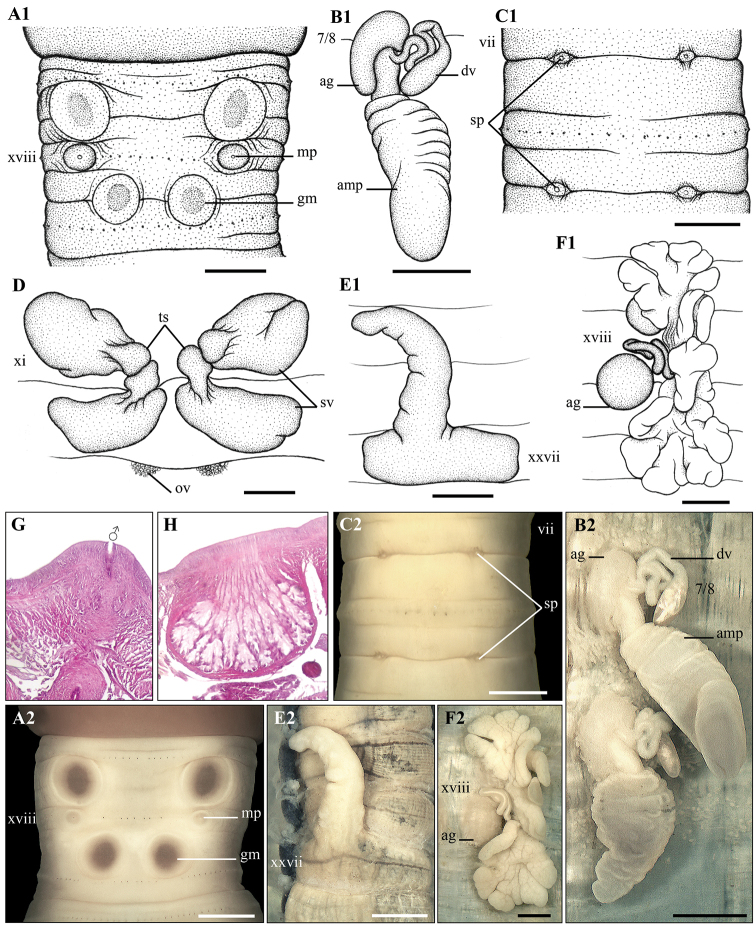
Variation of genital markings in the male region of *A. phuquocensis* sp. nov.

**Figure 13.**
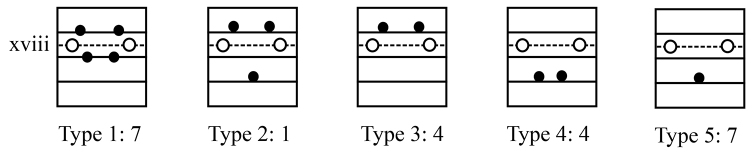
*A. phuquocsensis* sp. nov. Holotype (CTU–EW.190.h01) **A1, A2** Male region, ventral view **B1, B2** right spermathecae **C1, C2** spermathecal region, ventral view **D** testis sacs and vesicles **E** intestinal caecum **F1, F2** right prostatic gland **G** transection via male porophore **H** transection via genital marking. Scale bar: 1mm.

Figs 12 , 13 , Tables 2 , 3

####### Material examined.

***Holotype***: 1 mature (CTU-EW.188.h01), Kien Giang Province, Phu Quoc island, natural forests (10°20'51.3"N, 103°58'58.0"E), 44.2 m, 7 November 2016, coll. Lam HD & Trinh TKB.

***Paratypes***: 9 matures (CTU-EW.188.p02) same data as for holotype.

***Non-types***: 9 matures (CTU-EW.188.03) same data as for holotype; 4 matures (CTU-EW.188.04), Kien Giang Province, Phu Quoc island, natural forests (10°20'50"N, 103°55'07"E), 57 m, 7 November 2016, coll. Lam HD & Trinh TKB.

####### Diagnosis.

Medium-sized, length 74–145 mm, average diameter 2.5–4.7 mm, segments 116–145. Prostomium epilobous. Two pairs of spermathecal pores in ventral intersegments 7/8/9. Male pores in xviii, without copulatory pouches. Two pairs of genital markings present in ventral 17/18 and 18/19. Intestinal caeca simple. Holandric. Testis sacs in xi, separated. Septa 8/9/10/11 absent.

####### Etymology.

“*phuquocensis*”, named for the type locality.

####### Description.

Body cylindrical, medium size, length 74–145 mm, average diameter 2.5–4.7 mm, segments 116–145, weight 0.6–3.7 g. Body uniformly greyish brown except darkish brown clitellum. Prostomium 2/3 epilobous. First dorsal pore in 12/13. Pre-clitellum setae stouter and sparser than post-clitellum ones; setal numbers: 42–61 in viii, 50–77 in xxx, 5–9 between male porophores in xviii; setal distance: aa > ab, zz > zy. Clitellum within xiv–xvi, without setae and dorsal pores. Female pore single, in mid-ventral xiv.

Two pairs of spermathecal pores in ventral intersegments 7/8/9. Genital markings absent in the spermathecal region. Male pores located in the setal ring xviii, without copulatory pouches; ventral distance between two male pores about 0.28× body circumference. Genital markings roundly pad-shaped and variable in the male region, normally with two pairs in 17/18 and 18/19, or a pair in xix (near 19/20).

Septa 5/6/7/8 thick, 8/9/10/11 absent, 11/12/13 thick. Oesophageal gizzard within viii–xi. Intestinal origin at xv. Intestinal caeca simple, within xxvii–xxiv. Last hearts in xiii. Pharyngeal micronephridia in 5/6/7. Typhlosole lamelliform. Lymph lobuled, from 15/16.

Two pairs of spermathecae in viii and ix. Ampulla large, heart-shaped with transverse wrinkles; ducts short and stout. Diverticula waved, attached to the base of ampulla; seminal chamber ellipsoid. Accessory glands bean-shaped, surrounding spermathecal ducts.

Holandric. Testis sacs in xi, separated, the anterior pair poorly developed. Seminal vesicles well developed in xi and xii. Ovaries in 12/13. Ovisacs invisible. Prostate glands deeply lobuled in xvi–xx; ducts strongly coiled. Accessory glands present.

####### Habitats.

The species was occasionally found in natural forests along the main road to the northern part of Phu Quoc island. They were collected in the upper soil layer (0–10cm).

####### Vietnamese name.

Giun phú quốc.

####### Variations.

The body size and number of genital markings in the male region are variable among collected specimens. Specimens can be divided into two groups based on body size. The group of smaller specimens (*n* = 15) present the following ranges: length = 71–85 mm, diameter = 2.5–2.9 mm, segments = 116–145. The larger specimens (*n* = 8) present: length = 96–145 mm, diameter = 3.1–4.7 mm, segments = 124–145. Both two groups were together at the same site.

Genital markings are also variable in the male region. Genital markings have two pairs located in 17/18 and 18/19 (Type 1), but reduced to three genital markings (Type 2), one pair either in xvii or in xiv (Type 3 or Type 4), or a single one in xix (Type 5) (Fig. 12).

####### Remarks.

The new species can be assigned into the *Amynthas aeruginosus* group, which is characterized by having two pairs of spermathecal pores in 7/8/9 and holandric (Sims and Easton 1972). This group is known to have about 70 species distributed mainly in Southeast and East Asia. Of these, about 11 species have been recorded in Vietnam (Nguyen et al. 2016).

Within the *A. aeruginosus* group, the new species is fairly similar to *A. phimpheti* Hong, Inkavilay & James, 2018 and *A. antethecus* Hong, Inkavilay & James, 2018 in having two pairs of genital markings in the male region, but absent in the spermathecal region, and a simple intestinal caeca. However, these species can be distinguished by the positions of the first dorsal pore and the spermathecal pores and genital markings in the male region, the presence of septum 10/11, presence of accessory glands, and position of testis sacs (summarized in Table 2).

Regarding the arrangement of genital markings in the male region, the new species is slightly similar to *A. platycorpus* (Thai, 1982) and *A. binhgiaensis* (Le, 1994) by having two pairs of genital markings in 17/18 and 18/19. However, these species can be distinguished by number and position of spermathecal pores, first dorsal pore, genital markings in the spermathecal region, presence of septum 10/11, type of intestinal caeca and testis sacs (summarized in Table 3).

**Table 2.** Character comparison between *A. phuquocensis* sp. nov., *A. phimpheti* and *A. antethecus*.

Characters	*A. phuquocensis*	A. phimpheti^*^	A. antethecus^*^
Length (mm)	118–145	76–100	88–122
Diameter (mm)	3.7–4.7	2.2–4.8	3.0–4.1
Segments	143–145	99–104	82–109
Spermathecal pores	lateroventral	lateroventral	ventral
First dorsal pore	12/13	5/6	5/6
GM in male region	17/18 and 18/19 or xvii or xix	17/18 and 18/19	17/18 and xix
Septum 10/11	absent	present	present
Testis sacs	separated in xi	connected, x and xi	connected, x and xi
Accessory glands in spermathecal region	surrounding spermathecal ducts	no	no
Accessory glands in male region	present	absent	present

Source: *: Hong et al. (2018).

**Table 3.** Character comparison between *A. phuquocensis* sp. nov., *A. binhgiaensis*, *A. platycorpus*.

Characters	*A. phuquocensis*	*A. binhgiaensis* ^1^	*A. platycorpus* ^2^
Length (mm)	168–220	72	38–45
Diameter (mm)	6.50–7.58	3–4	2–3
Segments	99–133	100	40–51
Spermathecal pores	6/7/8/9, lateroventral	5/6/7/8, dorsal	6/7/8, lateral
First dorsal pore	12/13	12/13	4/5
GM in spermathecal region	4 pairs in vi–ix	absent	absent
Septum 10/11	absent	present	present
Intestinal caeca	lobuled	simple	manicate
Male sexual system	holandric	holandric	holandric
Testis sacs	xi, separated	x, xi, separated	x, xi, connected

Source: ^1^: Do et al. (1994); ^2^: Thai (1982).

###### Amynthas poropapillatus sp. nov.

Taxon classificationAnimaliaOpisthoporaMegascolecidae

2955009C-0F35-5B8A-83AF-D634C00747A8

http://zoobank.org/50D0E2DE-FC4F-4E9A-9B01-77653BB19FC9

Fig. 14 , Table 4

####### Material examined.

***Holotype***: 1C (CTU-EW.190.h01), Kien Giang Province, Phu Quoc island, natural forests (10°22'53"N, 104°00'22"E), 38 m, 7 November 2016, coll. Trinh TKB.

***Paratypes***: 9C (CTU-EW.190.p02) same data as for holotype.

***Non-types***: 11C (CTU-EW.190.03) same data as for holotype.

####### Diagnosis.

Medium-sized, length 139–170 mm, diameter 4.4–5.8 mm, segments 149–151. Prostomium epilobous. First dorsal pore in 12/13. Two pairs of spermathecal pores in ventral intersegments 7/8/9. Male pores located behind the setal ring xviii, on the posterior edge of genital markings. Two pairs of genital markings present in xvii and xviii. Intestinal caeca simple. Holandric. Testis sacs in xi, separated. Septa 8/9/10/11 absent.

####### Etymology.

“*poropapillatus*”, an adjective in apposition, to emphasize the position of male pores on the genital markings.

####### Description.

Body cylindrical, medium-sized, length 139–170 mm, diameter 4.4–5.8 mm, segments 149–151, weight 1.4–2.2 g. Body uniformly greyish brown except darkish brown clitellum. Prostomium 1/2 epilobous. First dorsal pore in 12/13. Pre-clitellum setae stouter and sparser than post-clitellum ones; setal numbers: 56–59 in viii, 84–85 in xxx, 17–20 between two male porophores in xviii; setal distance: aa = ab, zz = zy. Clitellum xiv–xvi, without setae and dorsal pores. Female pore single, in mid-ventral xiv.

Two pairs of spermathecal pores in intersegments 7/8/9. Male pores behind the setal ring xviii, on the posterior edge of genital markings, without copulatory pouches; ventral distance between two male pores about 0.33× circumference. Two pairs of genital markings present in xvii and xviii, slightly concave inside body wall.

Septa 5/6/7/8 thick, 8/9/10/11 absent, 11/12/13 thin. Oesophageal gizzard within viii–xi. Intestinal origin at xv. Intestinal caeca simple, within xxii–xxvii. Last hearts in xiii. Pharyngeal micronephridia in 5/6/7. Typhlosole lamelliform. Lymph glands lobuled, from 15/16.

Two pairs of spermathecae in viii and ix. Ampulla clavate, sometimes constricted; ducts short. Diverticula shorter than ampulla, folded several times, attached to the base of ampulla; seminal chamber oval. Each spermatheca with a bean-shaped accessory gland.

Holandric. Testis sacs in xi, separated. Seminal vesicles well developed in xi and xii. Ovaries in 12/13. Ovisacs invisible. Prostatic glands deeply lobuled in xvi–xxi; ducts long. Two pairs of accessory glands present.

####### Habitats.

The species was found only in natural forests in northern part of Phu Quoc island. It was collected in the upper soil layer (0–10 cm) of clay soils.

####### Vietnamese name.

Giun nhú phụ chứa lỗ đực.

####### Remarks.

The new species can be assigned to the *A. aeruginosus* group. Within the *A. aeruginosus* group, *A. poropapillatus* sp. nov. is similar to *A. nametensis* Hong, Inkhavilay & James, 2018 and *A. hoauykanangensis* Hong, Inkhavilay & James, 2018 in having the spermathecal pores located ventrally, the genital markings paired in xviii, the genital markings in spermathecal region absent, and a simple intestinal caeca. However, these three species can be distinguished by the first dorsal pore, number and position of genital markings in the male region, position of male pores, presence of septa 8/9 and 10/11, and intestinal origin (summarized in Table 4).

**Figure 14.**
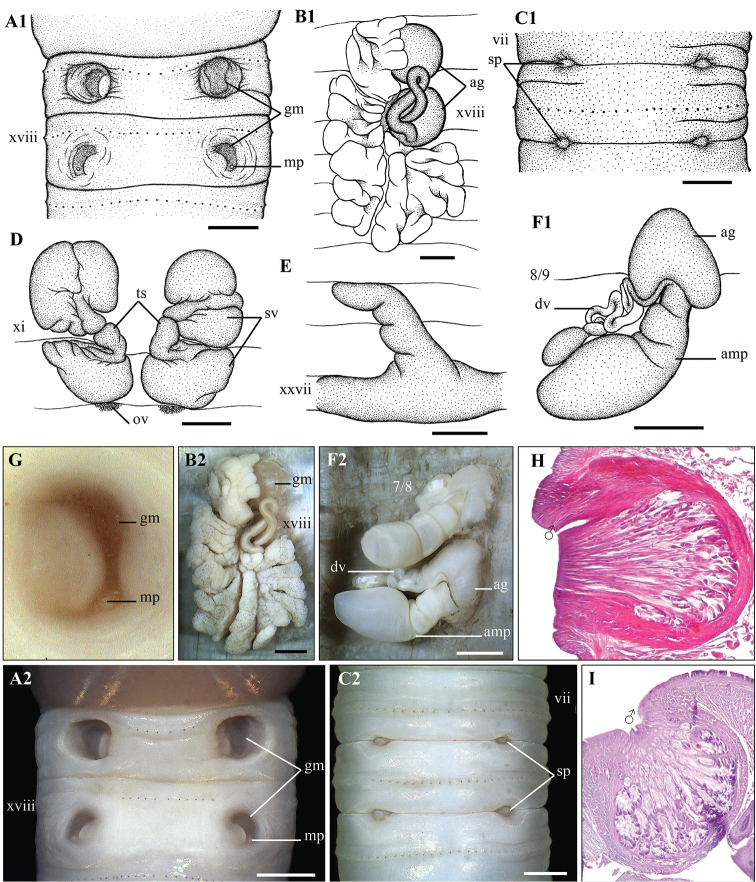
*Amynthas poropapillatus* sp. nov. Holotype (CTU–EW.190.h01) **A1, A2** male region, ventral view **B1, B2** right prostatic gland **C1, C2** spermathecal region, ventral view **D** testis sacs and vesicles **E** intestinal caecum **F1, F2** right spermathecae **G** male pore, ventral view **H** longitudinal transection via male porophore **I** transverse transection via male porophore. Scale bar: 1mm.

**Table 4.** Character comparison between *A. poropapillatus* sp. nov., *A. nametensis* và *A. hoauykanangensis*.

Characters	*A. poropapillatus*	A. nametensis^*^	A. hoauykanangensis^*^
Length (mm)	139–170	78–100	63–77
Diameter (mm)	4.4–5.8	4.5–6.0	3.0–4.0
Segments	149–151	121–136	78–101
First dorsal pore	12/13	12/13	4/5 or 5/6
Male pores	behind the setal ring xviii, on the posterior edge of genital marking	on the setal ring xviii	on the setal ring xviii
Ventral distance between two male pores	0.35	0.24–0.27	0.19–0.25
Genital markings in the male region	xvii, xviii	xviii, xix	xvii, xviii, xix
Septum 8/9	absent	thin	absent
Septum 10/11	absent	present	present
Testis sacs	xi	x, xi	x, xi
Intestinal origin	xv	xvi	xv
Accessory glands in spermathecal region	present	absent	absent
Accessory glands in male region	present	?	absent

Source: *: Hong et al. (2018).

##### Key to megascolecid species recorded in Phu Quoc island, southern Vietnam

**Table:** 

1	Clitellum xiv–xvii; each spermatheca with two diverticula	***Lampito mauritii***
–	Clitellum xiv–xvi; each spermatheca with a diverticulum	**2**
2	Copulatory pouches absent	**3**
–	Copulatory pouches present	**5**
3	Four pairs of spermathecal pores in 5/6/7/8/9. First dorsal pore in 5/6	***A. catenatus***
–	Two pairs of spermathecal pores in 7/8/9. First dorsal pore in 12/13	**4**
4	Male pores located behind the setal ring xviii. Genital markings strongly concave inside body wall in the male region	***A. poropapillatus***
–	Male pores in the setal ring xviii. Genital markings not concave	***A. phuquocensis***
5	Genital markings present in the male region	**6**
–	Genital markings absent in the male region	**9**
6	Four pairs of spermathecal pores in 5/6/7/8/9. Two pairs of genital markings in xvii and xix. Septum 8/9 thick	***M. posthuma***
–	Three pairs of spermathecal pores in 6/7/8/9. Two pairs of genital markings in 17/18 and 18/19. Septum 8/9 absent	**7**
7	Three to four pairs of genital markings in vi–ix. Septum 10/11 absent	***M. doiphamon***
–	No genital markings in the spermathecal region. Septum 10/11 present	**8**
8	Genital markings disc-shaped. Male region not concave	***M. peguana***
–	Genital markings slide-shaped. Male region concave	***M. bahli***
9	Two pairs of spermathecal pores in 6/7/8 or 7/8/9	**10**
–	Three pairs of genital markings in 6/7/8/9	**11**
10	Spermathecal pores in 6/7/8. First dorsal pore in 11/12	***M. planata***
–	Spermathecal pores in 7/8/9. First dorsal pore in 12/13	***M. californica***
11	Spermathecal pores located dorsally. First dorsal pore in 12/13	***M. dorsobitheca***
–	Spermathecal pores located lateroventrally. First dorsal pore in 11/12	***M. houlleti***


## Conclusion

As Phu Quoc island has a large area, the 12 species recorded at present may not reflect the true biodiversity of earthworms in this island. More intensive surveys likely will reveal additional new species and result in better understanding of the earthworm biodiversity of Vietnam.

## Supplementary Material

XML Treatment for Lampito mauritii

XML Treatment for Metaphire bahli

XML Treatment for Metaphire californica

XML Treatment for Metaphire doiphamon

XML Treatment for Metaphire houlleti

XML Treatment for Metaphire peguana

XML Treatment for Metaphire planata

XML Treatment for Metaph ire posthuma

XML Treatment for Metaphire dorsobitheca

XML Treatment for Amynthas catenatus

XML Treatment for Amynthas phuquocensis

XML Treatment for Amynthas poropapillatus
